# Covariation of the Incidence of Type 1 Diabetes with Country Characteristics Available in Public Databases

**DOI:** 10.1371/journal.pone.0118298

**Published:** 2015-02-23

**Authors:** Paula Andrea Diaz-Valencia, Pierre Bougnères, Alain-Jacques Valleron

**Affiliations:** 1 Institut National de la Santé et de la Recherche Médicale, Inserm Unité-1169, F-94276, Le Kremlin Bicêtre, France; 2 Université Pierre et Marie Curie-Paris 6, Ecole Doctorale 393, F-75012, Paris, France; 3 Assistance Publique-Hôpitaux de Paris, Hôpital Kremlin Bicêtre, Service Endocrinologie, F-94276, Le Kremlin Bicêtre, France

## Abstract

**Background:**

The incidence of Type 1 Diabetes (T1D) in children varies dramatically between countries. Part of the explanation must be sought in environmental factors. Increasingly, public databases provide information on country-to-country environmental differences.

**Methods:**

Information on the incidence of T1D and country characteristics were searched for in the 194 World Health Organization (WHO) member countries. T1D incidence was extracted from a systematic literature review of all papers published between 1975 and 2014, including the 2013 update from the International Diabetes Federation. The information on country characteristics was searched in public databases. We considered all indicators with a plausible relation with T1D and those previously reported as correlated with T1D, and for which there was less than 5% missing values. This yielded 77 indicators. Four domains were explored: *Climate and environment, Demography, Economy*, and *Health Conditions*. Bonferroni correction to correct false discovery rate (FDR) was used in bivariate analyses. Stepwise multiple regressions, served to identify independent predictors of the geographical variation of T1D.

**Findings:**

T1D incidence was estimated for 80 WHO countries. Forty-one significant correlations between T1D and the selected indicators were found. Stepwise Multiple Linear Regressions performed in the four explored domains indicated that the percentages of variance explained by the indicators were respectively 35% for *Climate and environment*, 33% for *Demography*, 45% for *Economy*, and 46% for *Health conditions*, and 51% in the *Final model*, where all variables selected by domain were considered. Significant environmental predictors of the country-to-country variation of T1D incidence included UV radiation, number of mobile cellular subscriptions in the country, health expenditure per capita, hepatitis B immunization and mean body mass index (BMI).

**Conclusions:**

The increasing availability of public databases providing information in all global environmental domains should allow new analyses to identify further geographical, behavioral, social and economic factors, or indicators that point to latent causal factors of T1D.

## Introduction

It has long been noticed that the incidence of Type 1 Diabetes (T1D) is highly variable from one country to another. For example, the 62.42/100.000 persons/year incidence found in Finland [1] was 780-fold larger than the 0.08/100.000 persons/year incidence in Papua New Guinea [2]; differences in T1D incidence are also observed between countries where the health care systems are comparable. The variability of T1D incidence is even visible within countries; for example, in Italy, T1D incidence varied between 54.4/100.000 persons/year in Sardinia [3] and 4.4/100.000 persons/year in Lombardia [4]. The reason for these differences is not precisely known, but is most unlikely due to classification bias, as the disease cannot go untreated, and the diagnosis is relative easy to perform in children [5]. The country-to-country T1D variability is known to be partly explained by genetic variations. Indeed, HLA (human leukocyte antigen) and 33 other genes are associated with elevated risk of T1D (*T1Dbase* Version 4.18 updated on 30/9/2014 available at http://www.t1dbase.org) [5–11]. The genetic characteristics of several populations have been found to—at least partially—explain the level of their T1D incidence [12]. For example, the low incidence in Japan, and more generally in southeast Asia, was strongly associated with the absence of highly susceptible haplotypes, such as DRB1*03-DQB1*0201 and DRB1*04-DQB1*0302 found in Caucasian populations [13] or DRB1*030101-DQB1*0201 [14] found in Arab populations (Bahrainis, Lebanese, and Tunisians). Instead, the major susceptible HLA haplotypes in the Japanese and Korean populations were DRB1*0405-DQB1*0401 and DRB1*0901-DQB1*0303 [15].

Another peculiarity of T1D epidemiology is that a dramatic increase of the incidence (on average 3% per year [16]) was observed over the last decades in many countries, in particular European countries with previously low incidences. This increase cannot be explained by genetic factors, since the genetic structure of these countries cannot have varied greatly over such a short period of time. The reasons are more likely to be found in environmental factors (taking here environment broadly, as encompassing physical, chemical, social and life-style factors). However, no single environmental factor, or configuration of factors, that could explain the patterns of differences has ever been identified. More likely, there are complex networks of environmental causes, and of gene-environmental causes that remain to be discovered.

The search of genetic factors of T1D was facilitated during the last 10 years by the GWAS (genome-wide association studies) technology that replaced the gene candidate approaches and instead scanned the entire genome to find SNPs (Single-Nucleotide Polymorphisms) that were significantly associated with T1D [17]. The discovery of an SNP was not the discovery of a “gene”, but was a marker leading to the possible discovery of a gene. Here, we translate this data-driven approach to search for environmental markers related to variations of T1D incidence that might eventually lead to environmental causes, possibly in interaction with genetic factors. One could indeed expect that, in this age of information, plenty of environmental characteristics could be readily available, insofar as local and global organizations collect such data, and provide them free to researchers, with easy interface on the Internet. A limitation commonly advocated is that country statistics are in many cases of too low quality. However, for two related reasons, this argument does not hold, or will not hold for long: a) why collect, maintain and publish such statistics if they cannot be used by researchers? and b) how can one encourage a better quality for these statistics—meaning more resources devoted to them—if they are never used?

Here, we present the attempt we made to use open public data to identify climate and environmental, demographic, economic, and health characteristics correlated with variations in T1D incidence between countries.

## Material and Methods

### Incidence of T1D data by country

The country T1D incidence was obtained thanks to a systematic review following the PRISMA recommendations [18] (**S1 Checklist**). All relevant original papers published in English between 1975 and 2014 including reviews (Diamond [16] and Eurodiab studies [19–21], the International Diabetes Federation (IDF) atlas [22, 23]) were analyzed (see Flow diagram of the literature search in **S1 Fig.**, the full search strategy on **S1 Table**, and the list of selected publications in **S2 Table**), registration number in the International Prospective Register of Systematic Reviews (PROSPERO): CRD42012002369 (**S1 Protocol)**.

The T1D incidence value per 100.000 persons/year for individuals in the age group 0–14 years for both sexes used here for each country was obtained as follows. 1) When only one dataset was available for a given country, the figure obtained from this study was used, even if the study was not nationwide. 2) If more than one dataset was available including nationwide and local studies, the most recent published nationwide study in the country was used. 3) If only local studies in a single area within the country were available, the most recent dataset was selected. 4) If local studies reporting incidences were available for different areas in the country, the mean incidence after grouping the most recent published studies for each area was used.

### Country indicators

Three open public databases from the World Health Organization (WHO) [24], the United Nations (UN) [25] and the World bank (WB) [26] were used to search indicators in four domains: *Climate and environment* (land use and physical environment), *Demography, Economy* (including health resources and expenses), and *Health*. We only kept indicators with less than 5% missing values in the 80 countries for which we could estimate T1D incidence (see Results, below).

**Fig 1:**
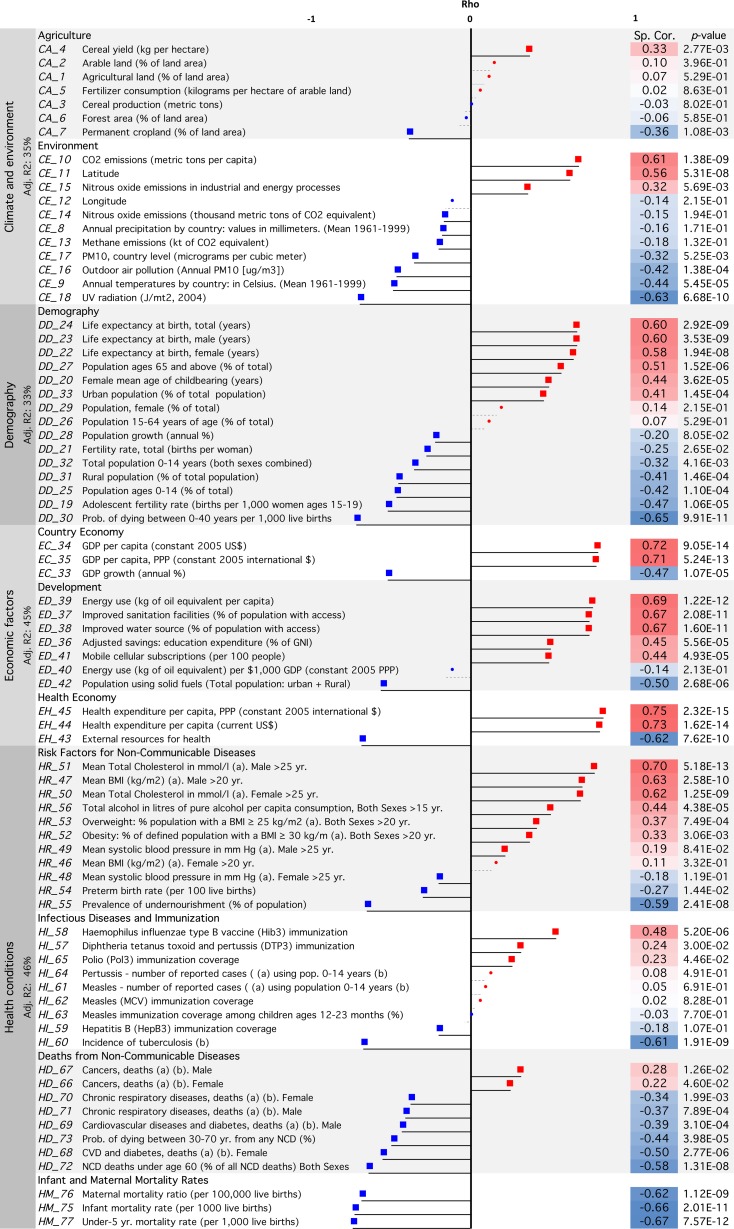
Correlations between T1D incidence and 77 country indicators. The correlations were computed in the 80 WHO countries where T1D incidence could be estimated. Dots: no significant correlations. Squares: significant correlations with *p≤*0.2. Significant correlations after Bonferroni correction (*p*-value ≤ 0.000649) are highlighted. Abbreviations: PM: particular matter, UV: ultraviolet, GDP: Gross Domestic Product, GNI: Gross National Income, BMI: Body Mass Index, CVD: Cardiovascular Diseases, CA: Cancer, DM: Diabetes Mellitus, CHRD: Chronic Respiratory Diseases, Adj. R2: Adjusted-R^2^. Red: positive correlations, blue: negative correlations. (a) Age-standardized estimate; (b) Per 100,000 individuals; (c) Coverage among 1-year-olds (%). See **S1 Database** for the entire database.

In total we used 77 indicators: two (latitude and longitude) were obtained thanks to Google Maps [27], 29 were retrieved from the WHO [24], three from the UN [25] and 43 from the WB [26]. When several values were available for one indicator, the value obtained in 2012, or on the date closest to 2012 was chosen (see Fig. 1 for a list of the 77 indicators, and **S1 Database** for the entire database used in the calculation of the correlations).

### Statistics

The R software (version 3.0.1) served for statistical and graphic analyses [28]. Spearman correlation was used to compute the correlation between the 77 selected country indicators and the T1D incidence. To account for multiple testing leading to a false discovery rate (FDR), the Bonferroni correction was used [29]: a *p*-value ≤ 0.000649 (= 0.05/77 variables) was considered as indicating significance at the 5% level.

We employed Stepwise Multiple Linear Regression (SMLR) methods to obtain the best predictors of T1D incidence within the original list of 77 variables. This process was carried out in two times: first, SMLR served to select independent predictors of T1D within each of the 4 environmental domains considered; second, the final subset of independent variables using the four sets of predictors obtained in the first step was selected.

In the by-domain analysis, all variables were entered for which the *p* value testing the correlation with incidence was smaller than 0.20. When a couple of variables was correlated with *r*>0.80, only one of the variables was used in the regression analysis to avoid computational issues associated with colinearity.

Then, starting with the full model, we made a manual backward selection of the variables, selecting at each step one variable. This variable was the one that, after being dropped, maximized the adjusted R^2^. The process was stopped at the step where the adjusted R^2^ decreased [30]. Then, taking the list of variables obtained, a new SMLR was performed that identified the smallest subset, based on the Akaike information criterion (AIC) [31]. Model assumptions for linear models were checked by visual inspection of the residuals.

The same process was used in the final analysis, starting with all significant predictors found in the analyses by domain. 10-Fold Cross-validation after bootstrapping [32] served to evaluate the predictive value of the final model. Graphic representation used the DAAG package [33].

## Results

Incidences were retrieved for 80 countries; they varied widely between continents, countries and regions (see **S2 Table** and **S1 Database** for the incidences). Among children aged 0–14 years the lowest nationwide incidences (≤ 1 per 100.000/year) were in Eastern Asia (China), South-East Asia (Thailand), Melanesia Oceania (Papua New Guinea), and South America and the Caribbean (Dominican Republic, and Paraguay). The highest nationwide incidences (≥ 30 per 100.000/year) were in Northern Europe (Finland, Sweden, Norway).

The correlations between the incidence and the 77 selected independent variables grouped into four domains are shown in Fig. 1 (for details and sources of information see **S1 Database**). Forty-one of 77 variables survived the Bonferroni correction and were significantly correlated with incidence at a significant *p*-value ≤ 0.000649 (= 0.05/77). Thirty-five variables, for which the correlation between any two of them was less than 0.8, and had a correlation coefficient with T1D incidence with *p* <0.20 were entered in the stepwise regression models.

**Fig 2:**
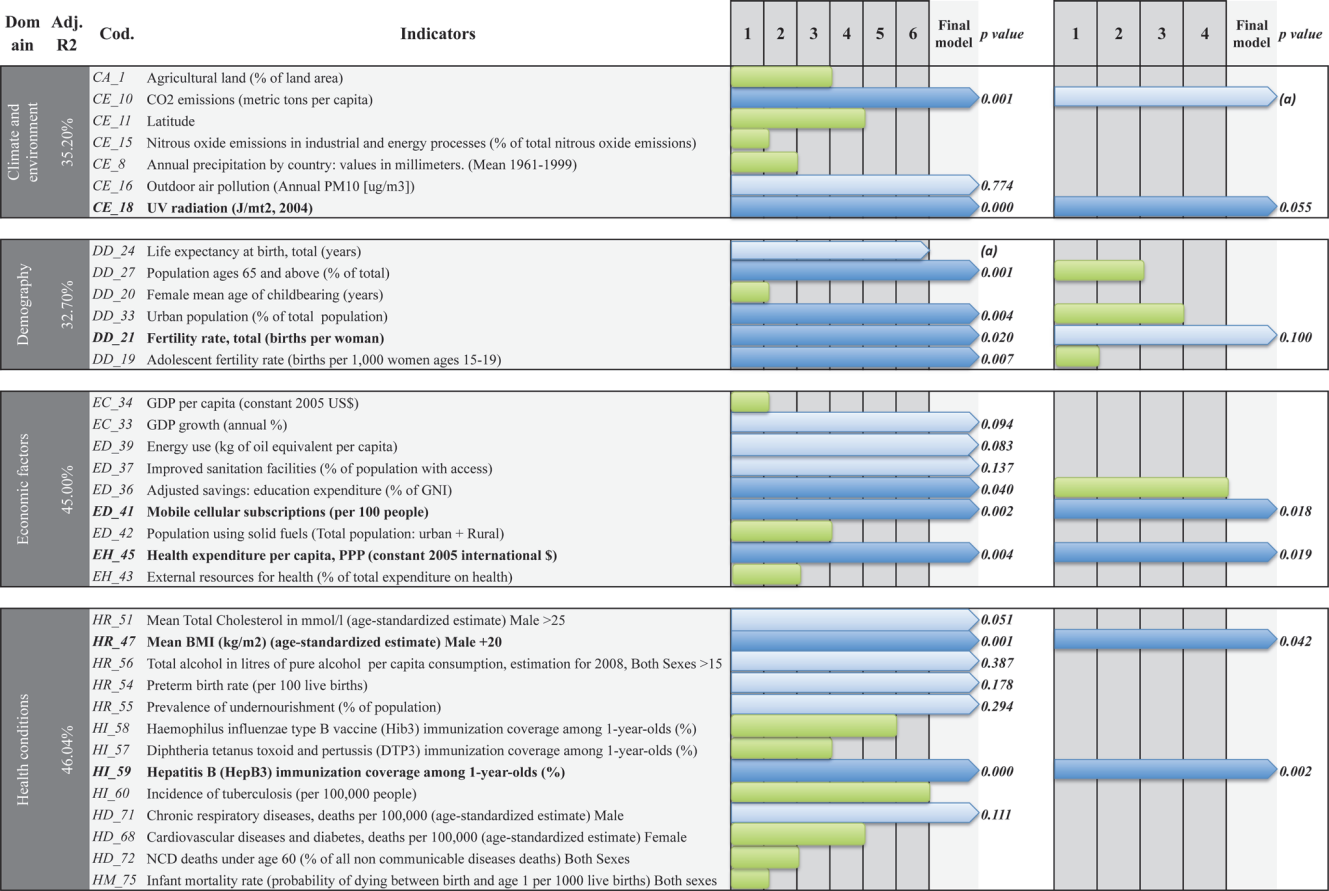
Stepwise identification of predictors of T1D Incidence (a) by domain and (b) final. The lines are the 35/77 variables with p<0.2. Green bars indicate variables that were excluded during the stepwise multiple regression (the length of the bar indicates the number of steps at which it was excluded). Blue bars indicate that variables were selected after SMLR was applied. The dark blue bars indicate that the independent predictors of T1D were highly significant; panel (A): in the by-domain analysis; panel (B): in the final model. The final analysis was performed on the variables selected in the by-domain analysis (shown in dark blue in panel (A). Abbreviations: Dom: Domain, PM: particular matter, UV: ultraviolet, GDP: Gross Domestic Product, BMI: Body Mass Index, CVD: Cardiovascular Diseases, CA: Cancer, DM: Diabetes Mellitus, CHRD: Chronic Respiratory Diseases, Adj.R2: Adjusted-R2. (a) Variable dropped after applied Akaike information criterion (AIC).

The detail of the 35 (of 77) variables that were entered in stepwise multiple regressions is shown in **Fig. 2 (panel A)** and the results of multivariate models by domains in **S3 Table**. In the *Climate and Environment* domain, seven of the 18 original variables qualified to be entered in the model; after the SLRM selection, the three selected variables were: UV radiation, CO_2_ emissions and outdoor air pollution (particular matter: PM10 μg/m^3^). The adjusted % of variance explained (R^2^) in the model was 35.2%. The four excluded variables were: % of agricultural land, latitude, nitrous oxide emissions and annual precipitations.

In the *Demography* domain, six of the 15 original variables qualified to be entered in the model; after the SLRM selection the four selected variables were: adolescent fertility rate, total fertility rate, proportion of the population aged over 65 years, and proportion of urban population; (adjusted R^2^ 32.7%). The two excluded variables were: life expectancy at birth, and female mean age of childbearing.

In the *Economy* domain, nine of the 13 original variables qualified to be entered in the model; after the SLRM selection the six selected variables were: energy use, proportion of mobile cellular subscriptions, education expenditure, % of improved sanitation facilities, health expenditure per capita, and % of annual Gross domestic product (GDP) growth; (adjusted R^2^ 45.0%). The three excluded variables were: GDP per capita, population using solid fuels and external resources for health.

In the *Health domain*, 13 of the 31 original variables qualified to be entered in the model; after the SLRM selection the seven selected variables were: deaths from chronic respiratory diseases, hepatitis B immunization coverage in <1 year of age, alcohol consumption, mean body mass index (BMI) in male >20, mean total cholesterol in male >25, preterm birth rate, and prevalence of undernourishment in the population (adjusted R^2^ 46%). The six excluded variables were: infant mortality rate, cardiovascular diseases and diabetes, deaths for non-communicable diseases ≤ 60, incidence of tuberculosis, haemophilus influenza type B (Hib3) vaccine immunization, and diphtheria tetanus toxoid and pertussis (DTP3) immunization.

**Fig 3:**
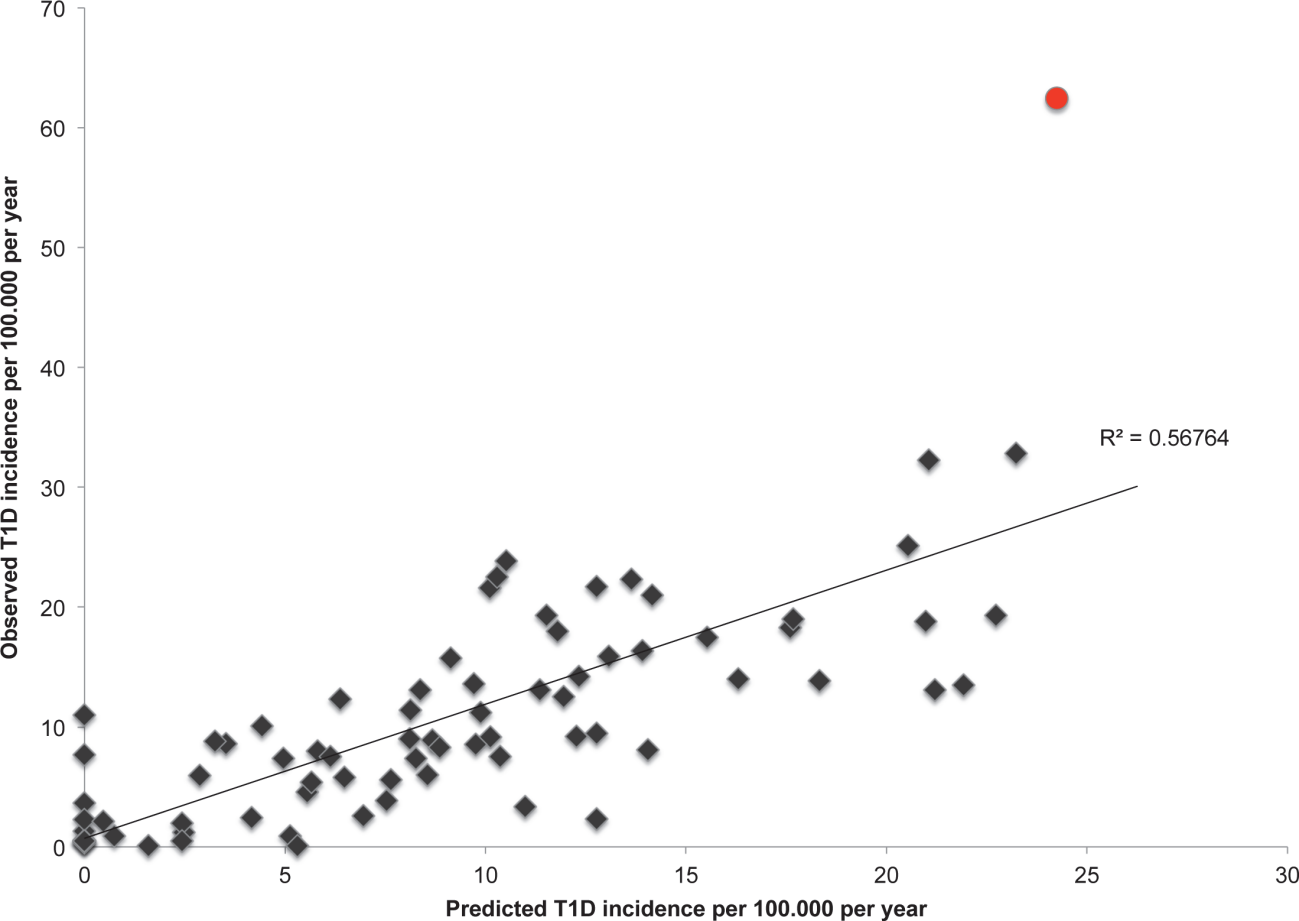
The predicted incidence of T1D among 80 countries vs. the observed incidence. Red dot: Finland. See S2 Fig. for predicted indices obtained after 10-fold Cross-validation.

In the final model, the five independent predictors were: UV radiation, number of mobile cellular subscriptions, health expenditure per capita, hepatitis B immunization and mean BMI (Table 1, details in **S3 Table**). The predicted incidence variation associated with the variation of each predictor can be computed from the values shown in Table 1: for example, for one unit increase in UV radiation in J/mt^2^, a mean 0.2% decrease in the incidence of T1D is expected. For an increase of 1% in the percentage of hepatitis B vaccination among 1 year-olds, an 8.5% decrease in the incidence of T1D is expected; for one unit increase in the percentage of BMI in males, a 1.3% increase in the incidence of T1D can be expected. The predictive value of the regression equation was visualized by comparing the predicted incidences with the observed incidences. Finland is noticeably an outlier in this empirical covariation predicted by the model (**Fig. 3**). 10-fold cross-validation indicated that the fraction of the variability of the global incidence of T1D was estimated to be 41% (**S2 Fig.**).

**Table 1:** Final Stepwise MLR model.

Code	Coefficients	Estimate	Std. Error	*p* Value
	(Intercept)	-25.240	15.390	0.106
**CE_18**	UV radiation (J/mt2, 2004)	-0.002	0.001	0.055
**DD_21**	Fertility rate, total (births per woman)	2.181	1.309	0.100
**ED_41**	Mobile cellular subscriptions (per 100 people)	0.066	0.027	0.018
**EH_45**	Health expenditure per capita (constant 2005 intern. $)	0.001	0.001	0.019
**HI_59**	Hepatitis B (HepB3) immunization coverage ^(a)^	-0.085	0.027	0.002
**HR_47**	Mean BMI (kg/m2) Male >20 years of age ^(b)^	1.263	0.611	0.042

The model included all significant predictors found in the analyses by domain.

(a) Among 1-year-olds (%)

(b) age-standardized estimate.

## Discussion

Our work explored whether the information available in public databases could help to study the environmental part of the country-to-country variations of T1D incidence. We found 77 variables with less than 5% missing values in the 80 countries for which the systematic review we carried out provided an estimate of the 0–14 T1D incidence.

These 77 variables explored four domains (*Climate and environment, Demography, Economy, Health*). In each of these domains, a stepwise multiple regression analyses identified the subset of variables of the domain which were independent predictors of the variation in the geographical T1D incidence. Then, analyzing these 4 subsets of domain variables, we identified five final independent environmental indicators: four of the five were known as risk factors of T1D: UV radiation [34–36], health expenditure per capita [34], coverage of hepatitis B vaccination [37] and mean BMI [38]. The last indicator was the proportion of mobile subscriptions, positively correlated with T1D incidence. It is probably a marker of a life-style that was not captured by any of the 77 variables used in the multiple analyses.

Coming to the domain-by-domain analysis **Fig. 2 (Panel B**), in the *Clim*a*te and Environment* domain, the first indicator that emerged was UV radiation. This may be related to vitamin D deficiency [39] as reported in previous studies [34]. The second indicator of this domain was CO_2_ emissions. An association between air pollution and T1D incidence has been recently described [40].

In the *Demography* domain, the only indicator that emerged in the final model was total fertility rate. The predictors dropped were: the adolescent fertility rate, the proportion of the population aged over 65 years, and the proportion of urban population. Concerning the proportion of urban population that was positively correlated with T1D incidence in this study as found in Lithuania [41], there are conflicting results in the literature: higher incidences was found in rural areas of the United Kingdom [42], and in semirural areas of Sweden [43], while no urban-rural difference in the incidence of T1D was found in Estonia [44].

In the *Economy* domain, three variables emerged, among which two were in the final list of independent predictors: proportion of mobile cellular subscriptions and health expenditure per capita. The third one was education expenditure. Higher incidences in wealthy countries, as measured by the GDP [45] and health expenditure per capita [34], have been described previously. In addition, other studies from Sweden [43, 46, 47], the United Kingdom [42, 48], and Canada [49] described similar associations between T1D and socioeconomic variables.

Within the *Health* domain, seven independent predictors were identified, among which two were in the final list: mean BMI in male >20, that had already been identified as risk factor [38], and hepatitis B immunization coverage in children <1 year of age, also recently proposed as a potential protector factor [37].

A first limitation of this study is that we could only rely on a relatively short set of variables retrieved in the public databases that met the condition of an acceptable fraction of missing data that we set arbitrarily at 5%. A second limitation was the differences in the temporality of the T1D data collected and the predictors. The mean year of the studies we used to estimate T1D incidence was 1997, while the mean year of the data for the 77 selected predictors was 2009. An important drawback, shared by all ecological studies, is that the correlations cannot be directly interpreted in terms of causality of the disease. However, they can be considered as providing signals that may help to unravel latent, yet unknown causes.

Furthermore, an important part of the variation in T1D incidence cannot be captured by an approach that only focuses on environmental factors. It is well known to be in a large part explained by genetic characteristics of the populations that were not analyzed here. Indeed, ethnic differences in T1D incidence between countries, and even within countries, have long been reported [13, 15, 50] and the most susceptible and protective haplotype determinants of T1D were identified [50]. However one difficulty is that this genetic information is lacking in many countries of the world, which limits the possibility of extending to genetics the kind of global analysis we presented here. For example, we explored the Allele Frequency Net at http://www.allelefrequencies.net/ [51], a public electronic repository for allele frequency of populations; we found that information on the haplotype DRB1*0405—DQA1*0301—DQB1*0302 for which the T1D Odd Ratio was extremely high (11.37 reported by the Type 1 Diabetes Genetics Consortium Families [50]) was available in only 6 countries. Moreover, the genetic characteristics of a country are much less homogeneous than the environmental characteristics, because of the presence of various ethnic groups within the same country, while many of the environmental variables that we studied can be considered as identical (latitude, geographical characteristics) in the subgroups of a country.

In the future, it can be expected that analyses similar to this one, taking advantage of the increased availability and quality of public databases characterizing the human populations would be a path to help identify some of the still unknown causes, and networks of causes, of TID.

## Supporting Information

S1 PRISMA Checklist CONSORT PRISMA checklist. (DOC)

S1 Fig Flow diagram literature search for T1D incidences. (TIFF)

S2 Fig 10-Fold Cross-Validation. (TIFF)

S1 Table Search strategy. (DOCX)

S2 Table Publications list of the incidence of T1D by country and area. Table showing the list of publications reporting T1D incidence used in the analyses (*). Information source: (PBDR) population based data register, (MBR) medical-based record, (OPD) other population denominators, (NS) non-specified. % Ascertainment: percentage of completeness between primary and secondary sources of registers. Data collection process reported in the article: (P) prospective—incident cases collected prospectively-, (H) historical-incident cases collected retrospectively-. First author and year of publication. Number of reference. NW: Nationwide study. Note: the age range for the reported incidence was 0–14 years, except in the following cases: (a) 0–12, (b) 0–13, (c) 0–18, (d) 0–19, (e) updated International Diabetes Federation (IDF) 2013.
(DOCX)

S3 Table Models by domains and final summary model. CI, Confidence Intervals. (a) Age-standardized estimate; (b) per 100,000 individuals; (c) Coverage among 1-year-olds (%).
(DOCX)

S1 Protocol PROSPERO protocol. International Prospective Register of Systematic Reviews.
(PDF)

S1 Database Database global incidence of T1D and independent variables. Excel table containing the database used in the correlations of 77 independent variables and the incidence of T1D among 80 countries.
(XLSX)
