# Supplementary figures and images for: Covariation of the Incidence of Type 1 Diabetes with Country Characteristics Available in Public Databases
Source: PLoS One. 2015 Feb 23;10(2):e0118298. doi: 10.1371/journal.pone.0118298 (PMC4338253; doi:10.1371/journal.pone.0118298)

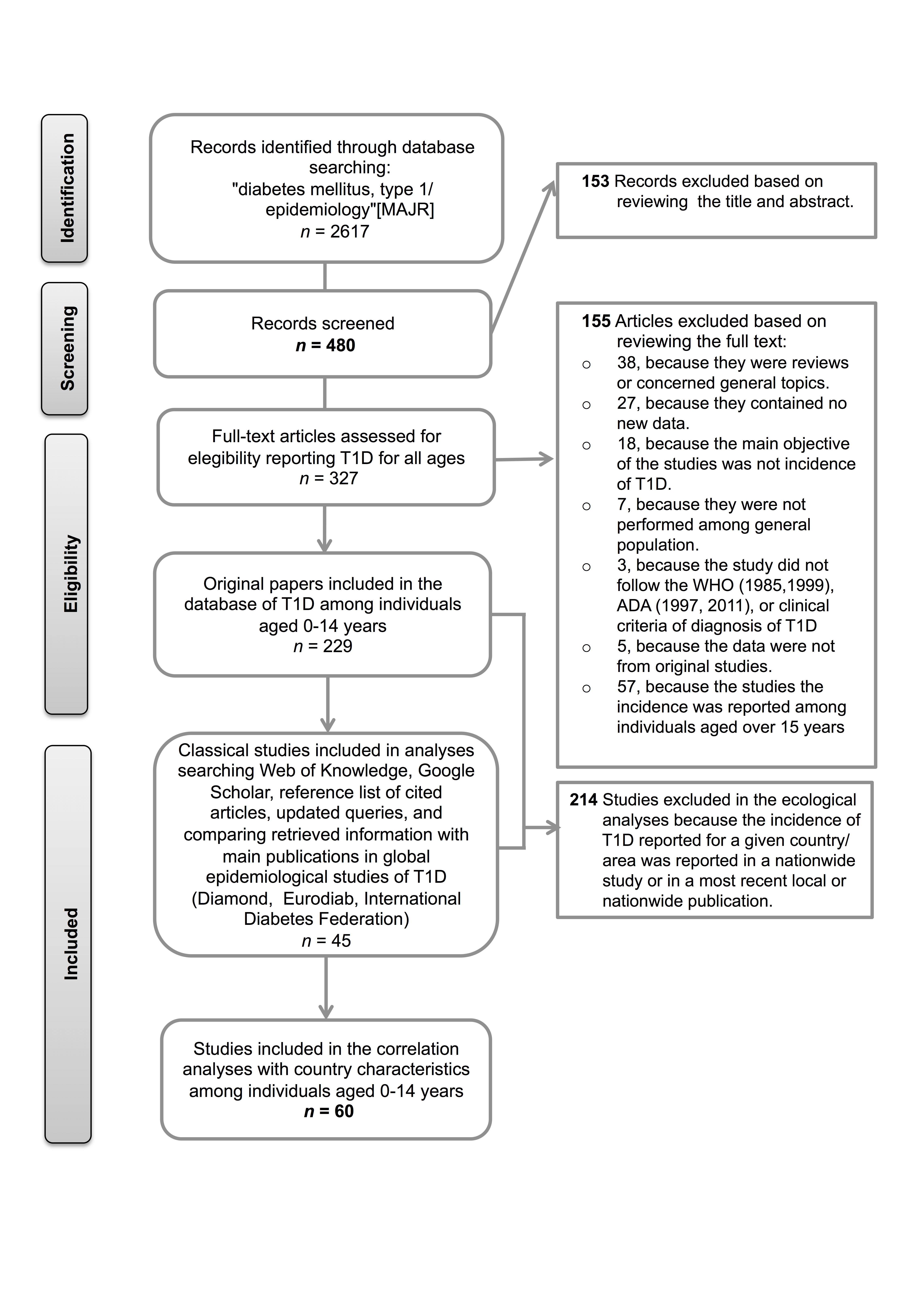

Supplement: S1 Fig — (TIFF) [file pone.0118298.s002.tiff]

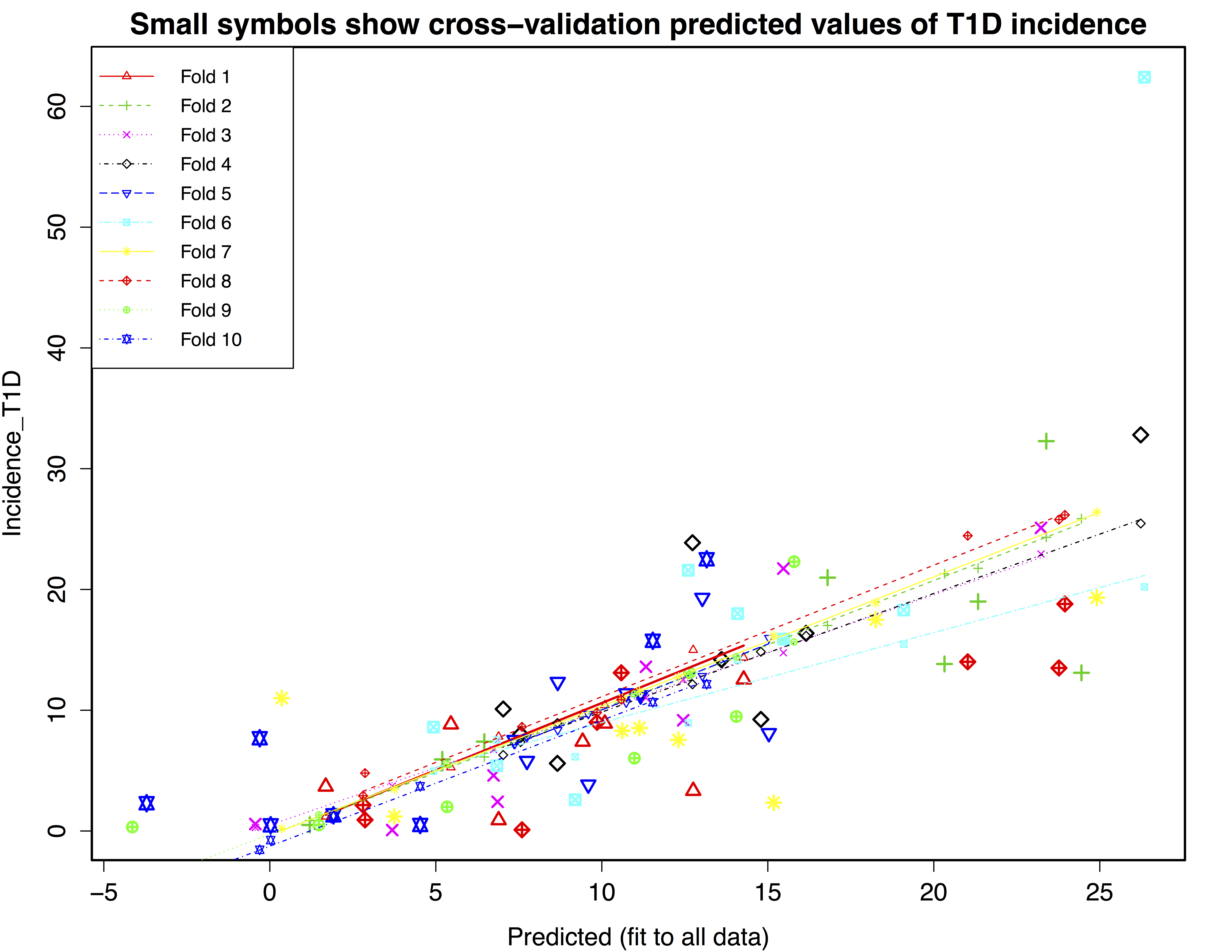

Supplement: S2 Fig — (TIFF) [file pone.0118298.s003.tiff]
