# Supplementary material for: Covariation of the Incidence of Type 1 Diabetes with Country Characteristics Available in Public Databases
Source: PLoS One. 2015 Feb 23;10(2):e0118298. doi: 10.1371/journal.pone.0118298 (PMC4338253; doi:10.1371/journal.pone.0118298)
Supplement: S1 Table — (DOCX) [file pone.0118298.s004.docx]

**Exploratory Search: 2011-11-28**

**PubMed** search equations:

*Search #1* ("diabetes mellitus, type 1/epidemiology" [Mesh Terms]) AND ("incidence" [MeSH Terms] OR "models, statistical"[MeSH Terms] OR cohort studies [MH])

*Search #2* ((trend [TI] AND incidence [TI]) OR (trends [TI] AND incidence [TI]) OR (onset [TI] AND Incidence [TI]) OR "birth cohort"[TIAB] OR "age period cohort"[TIAB] OR age-period-cohort [TIAB]) OR "incidence" [TIAB])

*Search #3* Journal Article [PT] NOT (Letter [PT] OR comment [PT] OR editorial [PT] OR news [PT]) AND ("1990/1/1" [PDAT]: "2011/11/28" [PDAT]). Limits: Humans.

At the end the equation was search ((*#1*) AND *#2*) AND *#3)*

**Web of Science** search equation:

*Topic* = (cohort studies OR (trend* OR incidence* OR tendency*) OR epidemiology*) AND

*Title* = ("type 1 diabetes" OR "IDDM" OR "childhood diabetes" OR "juvenile diabetes" OR "diabetes") AND

*Title* = ((trend AND incidence) OR (trends AND incidence) OR (onset AND Incidence) OR "birth cohort" OR "age period cohort" OR age-period-cohort).

The search was refined excluding: document type = (proceedings paper OR editorial material OR letter OR correction OR note). Databases=SCI-EXPANDED. Lemmatization=Off.

**Original Search: 2012-03-29**

After carried out the exploratory search 92 references were selected then we proposed a new search following 7 steps to get an optimal query:

**Step 1.** To perform an analysis of the MeSH terms presented in 92 selected articles after carried out the preliminary search equations.

**Step 2.** To carry out new queries in the MeSH Advanced Search Builder of PubMed^[[1]](#footnote-1)^ with an account of the main MeSH terms found in step 1.

**Step 3.** To check the MeSH terms for irrelevant references, so as to recognized the most common irrelevant MeSH terms.

**Step 4**. To introduce at the equation query of step 1 the most common MeSH terms found in irrelevant papers using the builder NOT to exclude irrelevant articles.

**Step 5.** To compare references linked in the different alternative queries. For example if we have two queries, query **A** and query **B**, using a strategy such as Venn diagrams (A∪B, A∩B), the references linked in the queries will be explored: query A, NOT query B, and query B, NOT query A. The first 60 references in each query will be examined: query A, query B, query A NOT B, and query B NOT A so as to analyze if they are relevant papers, and which one is the most accurate. To repeat steps 3 to 5 in the selected query until at least 50% (25/60) of the articles examined are pertinent. In this strategy we will identify the queries to which with a specific number can be assigned according to formulation of the queries.

**Step 6.** To examine the results of the selected query to verify that all 92 database references are included.

**Step 7.** Repeat steps 2 to 7 until a majority of 92 articles are included.

After repeating the step 2 to step 6, nine times we retrieve all the 92 articles included in the preliminary query in addition to new information.

| **Final Query Equation** | **No. References retrieved** |
| --- | --- |
| ("diabetes mellitus, type 1/epidemiology"[MAJR]) AND (("incidence"[MeSH Major Topic] OR incidence[Title/Abstract]) AND (age[Title/Abstract] OR "age distribution"[MeSH Terms] OR "age factors"[MeSH Terms] OR "age groups"[MeSH Terms] ) AND "humans"[MeSH Terms]) AND (Journal Article[ptyp] AND English[lang]) NOT ("Diabetic Neuropathies"[MeSH Terms] OR "Diabetic Nephropathies"[MeSH Terms] OR "Autoantibodies"[MeSH Terms] OR "Cholesterol"[MeSH Terms] OR "Antigens"[MeSH Terms] OR "histocompatibility antigens class ii"[MeSH Terms] OR "insulin"[MeSH Terms] OR "Vitamin D"[MeSH Terms] OR "Diabetic ketoacidosis"[MeSH Terms] OR "Vascular Diseases" [MeSH Terms] OR "Arteriosclerosis" [MeSH Terms] OR "Lipoproteins" [MeSH Terms] OR "Vascular Endothelial Growth Factors" [MeSH Terms] OR "Comorbidity" [MeSH Terms]) | 480 |

The final search equation was updated on 29 Apr. 2013 and 30 Jan. 2014.

**Glossary**

**For searching PubMed:**

**MeSH Major Topic [MAJR]:** a MeSH term that is one of the main topics discussed in the article denoted by an asterisk on the MeSH term or MeSH/Subheading combination, e.g., Cytokines/physiology* See MeSH Terms [MH] below.

**MeSH Subheadings [SH]:** MeSH [Subheadings](http://www.nlm.nih.gov.gate2.inist.fr/mesh/topsubscope.html) are used with MeSH terms to help describe more completely a particular aspect of a subject. For example, the drug therapy of asthma is displayed as asthma/drug therapy, see MeSH/Subheading Combinations in MeSH Terms [MH] below.

**MeSH Terms [MH]:** the NLM [Medical Subject Headings](http://www.nlm.nih.gov.gate2.inist.fr/mesh/meshhome.html) controlled vocabulary of biomedical terms that is used to describe the subject of each journal article in MEDLINE. MeSH terms are arranged hierarchically by subject categories with more specific terms arranged beneath broader terms. MeSH terms in PubMed automatically include the more specific MeSH terms in a search.

**Publication Type [PT]:** describes the type of material the article represents (e.g., Review, Clinical Trial, Retracted Publication, Letter); see the [PubMed Publication Types](http://www.ncbi.nlm.nih.gov.gate2.inist.fr/books/NBK3827/table/pubmedhelp.T42/?report=objectonly), e.g., review[pt].

**Title/Abstract [TIAB]:** words and numbers included in the title, abstract, and other abstract of a citation. English language abstracts are taken directly from the published article. If an article does not have a published abstract, NLM does not create one.

**Publication Date [DP]:** the date that the article was published.

Source and more details in: <http://www.ncbi.nlm.nih.gov.gate2.inist.fr/books/NBK3827/>

**For searching Web of Science:**

**SCI-EXPANDED:** Science Citation Index Expanded (--1975-present)

**SSCI:** Social Sciences Citation Index (--1975-present)

**A&HCI:** Arts & Humanities Citation Index (--1975-present)

**CPCI-S:** Conference Proceedings Citation Index- Science (--1990-present)

**CPCI-SSH:** Conference Proceedings Citation Index- Social Science & Humanities (--1990-present)

**Searching the Topic Field:**  enter Topic terms to search the following fields within a record: title, abstract, author keywords, Keywords Plus^®^. The product returns every record containing all your search terms.

**Searching the Title Field:** enter Title terms to limit your search to article titles.

**Lemmatization:** it finds (On) or not (Off) alternative forms of the search term, for example, tooth and teeth.

Source and more details in:

<http://images.webofknowledge.com.gate2.inist.fr/WOKRS55B6/help/WOS/contents.html>

1. <http://www.ncbi.nlm.nih.gov.gate2.inist.fr/mesh/advanced> [↑](#footnote-ref-1)
