# Supplementary material for: Covariation of the Incidence of Type 1 Diabetes with Country Characteristics Available in Public Databases
Source: PLoS One. 2015 Feb 23;10(2):e0118298. doi: 10.1371/journal.pone.0118298 (PMC4338253; doi:10.1371/journal.pone.0118298)
Supplement: S2 Table — Table showing the list of publications reporting T1D incidence used in the analyses (*). Information source: (PBDR) population based data register, (MBR) medical-based record, (OPD) other population denominators, (NS) non-specified. % Ascertainment: percentage of completeness between primary and secondary sources of registers. Data collection process reported in the article: (P) prospective—incident cases collected prospectively-, (H) historical-incident cases collected retrospectively-. First author and year of publication. Number of reference. NW: Nationwide study. Note: the age range for the reported incidence was 0–14 years, except in the following cases: (a) 0–12, (b) 0–13, (c) 0–18, (d) 0–19, (e) updated International Diabetes Federation (IDF) 2013. (DOCX) [file pone.0118298.s005.docx]

| **Country** | **Area** | **Study period** | **Mean T1D Inc*** | **Inc. by area** | **Ascertainment %** | **Info. Source** | **Data collection** | **Author, Pub yr.** | **Ref.** |
| --- | --- | --- | --- | --- | --- | --- | --- | --- | --- |
| **Algeria** | |  |  |  |  |  |  |  |  |
|  | Oran | 1990_1999 | **8.60** | **8.60** | NA | PBDR | P or H | Diamond, 2006 | [1] |
| **Argentina** | |  |  |  |  |  |  |  |  |
|  | Cordoba | 1991_1992 | **7.55** | **7.00** | 90.0 | PBDR | P or H | Diamond, 2006 | [1] |
|  | Avellaneda | 1990_1996 | **7.55** | **6.30** | 94.0 | PBDR | P or H | Diamond, 2006 | [1] |
|  | Tierra del Fuego | 1993_1996 | **7.55** | **10.30** | 100.0 | PBDR | P or H | Diamond, 2006 | [1] |
|  | Corrientes | 1992_1999 | **7.55** | **6.60** | 95.0 | PBDR | P or H | Diamond, 2006 | [1] |
| **Australia** | |  |  |  |  |  |  |  |  |
|  | NW | 2000_2006 | **22.50** | **22.50** | 97.1 | PBDR | P | Catanzariti L, 2009 | [2] |
| **Austria** | |  |  |  |  |  |  |  |  |
|  | NW | 2004_2008 | **17.50** | **17.50** | 97.2 | PBDR | P | Patterson C, 2012 | [3] |
| **Bahamas** | |  |  |  |  |  |  |  |  |
|  | NW | 2001_2002 | **10.10** | **10.10** | NA | MBR | P | Peter S A, 2005 | [4] |
| **Barbados** | |  |  |  |  |  |  |  |  |
|  | NW | 1990_1993 | **2.00** | **2.00** | NA | PBDR | P or H | Diamond, 2006 | [1] |
| **Belarus** | |  |  |  |  |  |  |  |  |
|  | Gomel, Minsk | 1997_2002 | **5.60** | **5.60** | 100.0 | PBDR | NA | Zalutskaya A, 2004 | [5] |
| **Belgium** | |  |  |  |  |  |  |  |  |
|  | Antwerp | 2004_2008 | **15.90** | **15.90** | 94.9 | PBDR | P | Patterson C, 2012 | [3] |
| **Bosnia and Herzegovina** | |  |  |  |  |  |  |  |  |
|  | Tuzla Canton | 1995_2004 | **7.53** | **6.93** | 100.0 | PBDR | P | Tahirovic H, 2007 | [6] |
|  | Republic of Srpska | 1998_2010 | **7.53** | **8.13** | 100.0 | PBDR | P | Radosevic B, 2013 | [7] |
| **Brazil** | |  |  |  |  |  |  |  |  |
|  | Sao Paulo (Bauru) | 1986_2006 | **8.83** | **10.66** | 94.7 | PBDR | P | Negrato C, 2010 | [8] |
|  | Rio Grande do Sul (Passo Fundo) | 1996_1999 | **8.83** | **7.00** | 82.5 | PBDR | P or H | Diamond, 2006 | [1] |
| **Bulgaria** | |  |  |  |  |  |  |  |  |
|  | Eastern | 1989_1994 | **8.53** | **6.80** | 99.9 | PBDR | P | Eurodiab, 2000 | [9] |
|  | Varna | 1990_1999 | **8.53** | **8.10** | 100.0 | PBDR | P or H | Diamond, 2006 | [1] |
|  | Western | 1990_1999 | **8.53** | **10.70** | 99.5 | PBDR | P or H | Diamond, 2006 | [1] |
| **Canada** | |  |  |  |  |  |  |  |  |
|  | Toronto ^(^**^c)^** | 1976_1978 | **21.73** | **9.00** | 97.2 | PBDR | P, H | Ehrlich R M, 1982 | [10] |
|  | Manitoba | 1985_1993 | **21.73** | **20.65** | 95.0 | PBDR | P | Blanchard J F, 1997 | [11] |
|  | Prince Edward Island | 1990_1993 | **21.73** | **24.50** | 100.0 | PBDR | P or H | Diamond, 2006 | [1] |
|  | Alberta (Edmonton) | 1990_1996 | **21.73** | **23.30** | 85.5 | PBDR | P or H | Diamond, 2006 | [1] |
|  | Calgary | 1990_1999 | **21.73** | **20.60** | 100.0 | PBDR | P or H | Diamond, 2006 | [1] |
|  | Québec | 1989_2000 | **21.73** | **15.34** | NA | PBDR | P | Legault L, 2006 | [12] |
|  | Newfoundland and Labrador | 1987_2010 | **21.73** | **38.68** | NA | PBDR | P, H | Newhook L A, 2012 | [13] |
| **Chile** | |  |  |  |  |  |  |  |  |
|  | IX Region | 1980_1993 | **3.84** | **1.37** | 97.0 | PBDR | P | Larenas G, 1996 | [14] |
|  | Santiago of Chile (Communes of Metropolitan region) | 2000_2005 | **3.84** | **6.30** | 100.0 | PBDR | P | Torres-Avilés F, 2010 | [15] |
|  | NW | 1988_1994 | **0.47** | **0.47** | 93.0 | PBDR | P, H | Yang Z, 2005 | [16] |
| **Colombia** | |  |  |  |  |  |  |  |  |
|  | Bogota | 1990_1990 | **2.15** | **3.80** | 97.0 | PBDR | P or H | Diamond, 2006 | [1] |
|  | Cali | 1995_1999 | **2.15** | **0.50** | NA | PBDR | P or H | Diamond, 2006 | [1] |
| **Croatia** | |  |  |  |  |  |  |  |  |
|  | NW | 1995_2003 | **8.90** | **8.90** | 98.5 | PBDR | P | Stipanic G, 2012 | [17] |
| **Cuba** | |  |  |  |  |  |  |  |  |
|  | NW | 1990_1999 | **2.30** | **2.30** | 62.5 | PBDR | P or H | Diamond, 2006 | [1] |
| **Cyprus** | |  |  |  |  |  |  |  |  |
|  | NW | 1990_2009 | **12.34** | **12.34** | 50.0 | PBDR | NA | Skordis N, 2012 | [18] |
| **Czech Republic** | |  |  |  |  |  |  |  |  |
|  | NW | 2004_2008 | **19.30** | **19.30** | 97.4 | PBDR | P | Patterson C, 2012 | [3] |
| **Dem. People's Republic of Korea** | | |  |  |  |  |  |  |  |
|  | Seoul | 1990_1991 | **1.10** | **1.10** | NA | PBDR | P or H | Diamond, 2006 | [1] |
| **Denmark** | |  |  |  |  |  |  |  |  |
|  | NW | 2004_2008 | **25.10** | **25.10** | 99.2 | PBDR | P | Patterson C, 2012 | [3] |
| **Dominican Republic** | |  |  |  |  |  |  |  |  |
|  | NW | 1995_1999 | **0.50** | **0.50** | 53.0 | PBDR | P or H | Diamond, 2006 | [1] |
| **Egypt** | |  |  |  |  |  |  |  |  |
|  | Alexandria, Damanhour | 1992_1992 | **8.00** | **8.00** | NA | OPD | NA | Arab M, 1992 | [19] |
| **Estonia** | |  |  |  |  |  |  |  |  |
|  | NW | 1983_2006 | **13.09** | **13.09** | 98.0 | PBDR | P, H | Teeaar T, 2010 | [20] |
| **Ethiopia** | |  |  |  |  |  |  |  |  |
|  | Gondar | 1995_2008 | **0.33** | **0.33** | NA | MBR | P | Alemu S, 2009 | [21] |
|  | Jimma | 2002_2008 | **0.33** | **0.33** | NA | MBR | P | Alemu S, 2009 | [21] |
| **Finland** | |  |  |  |  |  |  |  |  |
|  | NW | 2006_2011 | **62.42** | **62.42** | NS | OPD | NA | Harjutsalo V, 2013 | [22] |
| **France** | |  |  |  |  |  |  |  |  |
|  | Franche-Comté | 1980_1998 | **9.24** | **7.01** | 80.6 | PBDR | H | Mauny F, 2005 | [23] |
|  | Aquitanie, Lorraine, Normandia Basse, Normandia Haut | 1990_1994 | **9.24** | **8.50** | 97.0 | PBDR | P or H | Diamond, 2006 | [1] |
|  | Aquitaine | 1998_2004 | **9.24** | **12.20** | NA | OPD | NA | Barat P, 2008 | [24] |
| **Georgia** | |  |  |  |  |  |  |  |  |
|  | NW | 1998_1999 | **4.60** | **4.60** | NA | OPD | NA | Amirkhanashvili, 2000 | [25] |
| **Germany** | |  |  |  |  |  |  |  |  |
|  | Düsselforf | 1999_2003 | **20.98** | **18.30** | 95.4 | PBDR | P | Patterson C, 2009 | [26] |
|  | Baden-Württemberg | 2004_2007 | **20.98** | **21.80** | 100.0 | PBDR | P | Patterson C, 2012 | [3] |
|  | North Rhine-Westphalia | 2004_2008 | **20.98** | **23.70** | 98.6 | PBDR | P | Patterson C, 2012 | [3] |
|  | Saxony | 2004_2008 | **20.98** | **20.10** | 93.6 | PBDR | P | Patterson C, 2012 | [3] |
| **Greece** | |  |  |  |  |  |  |  |  |
|  | NW | 1992_1992 | **6.03** | **6.03** | NA | PBDR | P | Dacou-Voutetakis C, 1995 | [27] |
| **Hungary** | |  |  |  |  |  |  |  |  |
|  | 18 of 19 countries (All, less Budapest) | 2004_2008 | **18.30** | **18.30** | 98.7 | PBDR | P | Patterson C, 2012 | [3] |
| **Iceland** | |  |  |  |  |  |  |  |  |
|  | NW | 1989_1994 | **13.50** | **13.50** | 100.0 | PBDR | P | Eurodiab, 2000 | [9] |
| **India** | |  |  |  |  |  |  |  |  |
|  | Madras | 1991_1994 | **11.00** | **11.00** | 90.0 | PBDR | H | Ramachandran A, 1996 | [28] |
| **Iran (Islamic Republic of)** | |  |  |  |  |  |  |  |  |
|  | Fars | 1991_1996 | **3.68** | **3.68** | 100.0 | PBDR | P | Pishdad G R, 2005 | [29] |
| **Ireland** | |  |  |  |  |  |  |  |  |
|  | NW | 1997_1997 | **16.37** | **16.37** | 90.6 | PBDR | P | Roche E F, 2002 | [30] |
| **Israel** | |  |  |  |  |  |  |  |  |
|  | NW: Population: Arabs | 1997_2003 | **9.17** | **7.59** | NA | PBDR | P | Koton S, 2007 | [31] |
|  | NW: Population: Jews and others | 1997_2003 | **9.17** | **10.76** | NA | PBDR | P | Koton S, 2007 | [31] |
| **Italy** | |  |  |  |  |  |  |  |  |
|  | NW_39.7% population | 1990_2003 | **12.55** | **12.55** | NA | PBDR | P | Bruno G, 2010 | [32] |
| **Japan** | |  |  |  |  |  |  |  |  |
|  | NW | 1998_2001 | **2.35** | **2.35** | NA | PBDR | P | Kawasaki E, 2006 | [33] |
| **Jordan** | |  |  |  |  |  |  |  |  |
|  | NW | 1992_1996 | **3.33** | **3.33** | 95.0 | NS | P, H | Ajlouni K, 1999 | [34] |
| **Kuwait** | |  |  |  |  |  |  |  |  |
|  | NW | 1992_1999 | **22.30** | **22.30** | 87.5 | PBDR | P or H | Diamond, 2006 | [1] |
| **Latvia** | |  |  |  |  |  |  |  |  |
|  | NW | 1990_1999 | **7.40** | **7.40** | NA | PBDR | P or H | Diamond, 2006 | [1] |
| **Libyan Arab Jamahiriya** | |  |  |  |  |  |  |  |  |
|  | Benghazi | 1991_1999 | **9.00** | **9.00** | NA | PBDR | P or H | Diamond, 2006 | [1] |
| **Lithuania** | |  |  |  |  |  |  |  |  |
|  | NW | 2004_2008 | **14.20** | **14.20** | NA | PBDR | P | Patterson C, 2012 | [3] |
| **Luxembourg** | |  |  |  |  |  |  |  |  |
|  | NW | 2004_2008 | **19.00** | **19.00** | 100.0 | PBDR | P | Patterson C, 2012 | [3] |
| **Malta** | |  |  |  |  |  |  |  |  |
|  | NW | 2006_2010 | **23.87** | **23.87** | 100.0 | PBDR | P | Formosa N, 2012 | [35] |
| **Mauritius** | |  |  |  |  |  |  |  |  |
|  | NW | 1990_1994 | **1.30** | **1.30** | 67.5 | PBDR | P or H | Diamond, 2006 | [1] |
| **Mexico** | |  |  |  |  |  |  |  |  |
|  | NW ^(^**^d)^** | 2000_2010 | **5.93** | **5.93** | NA | PBDR | H | Gomez-Diaz RA, 2012 | [36] |
| **Montenegro** | |  |  |  |  |  |  |  |  |
|  | NW | 2004_2008 | **17.50** | **17.50** | 100.0 | PBDR | P | Patterson C, 2012 | [3] |
| **Netherlands** | |  |  |  |  |  |  |  |  |
|  | NW | 1996_1999 | **18.80** | **18.80** | NA | OPD | NA | Van Wouwe J P, 2002 | [37] |
| **New Zealand** | |  |  |  |  |  |  |  |  |
|  | NW | 1999_2000 | **18.00** | **18.00** | 95.0 | PBDR | NA | Campbell-Stokes PL, 2005 | [38] |
| **Norway** | |  |  |  |  |  |  |  |  |
|  | NW | 2004_2008 | **32.80** | **32.80** | 92.0 | PBDR | P | Patterson C, 2012 | [3] |
| **Oman** | |  |  |  |  |  |  |  |  |
|  | NW | 1993_1995 | **2.59** | **2.59** | 96.0 | PBDR | P | Soliman A T, 1996 | [39] |
| **Pakistan** | |  |  |  |  |  |  |  |  |
|  | Karachi | 1990_1999 | **0.50** | **0.50** | 51.0 | PBDR | P or H | Diamond, 2006 | [1] |
| **Papua New Guinea** | |  |  |  |  |  |  |  |  |
|  | NW | 1996_2000 | **0.08** | **0.08** | NA | MBR | P | Ogle, 2001 | [40] |
| **Paraguay** | |  |  |  |  |  |  |  |  |
|  | NW | 1990_1999 | **0.90** | **0.90** | NA | PBDR | P or H | Diamond, 2006 | [1] |
| **Peru** | |  |  |  |  |  |  |  |  |
|  | Lima | 1990_1994 | **0.50** | **0.50** | 67.5 | PBDR | P or H | Diamond, 2006 | [1] |
| **Poland** | |  |  |  |  |  |  |  |  |
|  | NW | 1989_2004 | **11.23** | **11.23** | NA | PBDR | P | Jarosz-Chobot P, 2011 | [41] |
| **Portugal** | |  |  |  |  |  |  |  |  |
|  | Algarve | 1990_1994 | **13.10** | **14.60** | 87.0 | PBDR | P or H | Diamond, 2006 | [1] |
|  | Portalegre | 1990_1994 | **13.10** | **21.30** | 93.0 | PBDR | P or H | Diamond, 2006 | [1] |
|  | Coimbra | 1990_1999 | **13.10** | **9.60** | 100.0 | PBDR | P or H | Diamond, 2006 | [1] |
|  | Madeira Island | 1990_1999 | **13.10** | **6.90** | 100.0 | PBDR | P or H | Diamond, 2006 | [1] |
| **Qatar** | |  |  |  |  |  |  |  |  |
|  | NW | 1992_1996 | **11.40** | **11.40** | NA | OPD | NA | Al-Zyoud M, 1997 | [42] |
| **Romania** | |  |  |  |  |  |  |  |  |
|  | NW | 2000_2004 | **5.40** | **5.40** | NA | OPD | NA | Serban V, 2005 | [43] |
| **Russian Federation** | |  |  |  |  |  |  |  |  |
|  | Novosibirsk | 1990_1999 | **9.48** | **6.90** | 93.5 | PBDR | P or H | Diamond, 2006 | [1] |
|  | Moscow | 1996_2005 | **9.48** | **12.07** | 94.0 | PBDR | P | Pronina E A, 2008 | [44] |
| **Saudi Arabia** | |  |  |  |  |  |  |  |  |
|  | Eastern Province | 1986_1997 | **21.59** | **12.30** | 100.0 | PBDR | NA | Kulaylat N A, 2000 | [45] |
|  | Al-Madinah (North West) ^(a)^ | 2004_2009 | **21.59** | **30.88** | NA | PBDR | P | Habeb A M, 2011 | [46] |
| **Serbia** | |  |  |  |  |  |  |  |  |
|  | Belgrade ^(e)^ | 2000_2004 | **12.90** | **12.90** | NA | OPD | NA | Vlajinac H D, 1995 | [47] |
| **Singapore** | |  |  |  |  |  |  |  |  |
|  | NW ^(a)^ | 1992_1994 | **2.42** | **2.42** | 92.2 | PBDR | P | Lee WW, 1998 | [48] |
| **Slovakia** | |  |  |  |  |  |  |  |  |
|  | NW | 1999_2003 | **13.60** | **13.60** | 100.0 | PBDR | P | Patterson C, 2009 | [26] |
| **Slovenia** | |  |  |  |  |  |  |  |  |
|  | NW | 1998_2010 | **13.83** | **13.83** | 100.0 | PBDR | P | Radosevic B, 2013 | [7] |
| **Spain** | |  |  |  |  |  |  |  |  |
|  | Madrid | 1985_1988 | **15.75** | **10.60** | 90.0 | PBDR | H | Serrano Rios M, 1990 | [49] |
|  | Caceres ^(b)^ | 1988_1999 | **15.75** | **16.67** | 99.2 | PBDR | H | Lora-Gomez R E, 2005 | [50] |
|  | Badajoz | 1992_1996 | **15.75** | **17.23** | 95.0 | PBDR | P | Morales-Perez F M, 2000 | [51] |
|  | Navarre | 2003_2004 | **15.75** | **22.17** | 99.6 | PBDR | P | Bahíllo M, 2007 |  |
|  | Catalonia | 2004_2008 | **15.75** | **12.10** | 97.6 | PBDR | P | Patterson C, 2012 | [3] |
| **Sudan** | |  |  |  |  |  |  |  |  |
|  | Gezira | 1990_1990 | **7.68** | **5.00** | 100.0 | PBDR | P or H | Diamond, 2006 | [1] |
|  | Khartoum | 1991_1995 | **7.68** | **10.10** | 97.0 | PBDR | NA | Elamin et al, 1997 | [52] |
| **Sweden** | |  |  |  |  |  |  |  |  |
|  | NW | 1983_2007 | **32.28** | **32.28** | 98.0 | PBDR | P | Dahlquist G G, 2011 | [53] |
| **Switzerland** | |  |  |  |  |  |  |  |  |
|  | NW | 2004_2008 | **13.10** | **13.10** | 91.3 | PBDR | P | Patterson C, 2012 | [3] |
| **TFYR Macedonia** | |  |  |  |  |  |  |  |  |
|  | NW | 2004_2008 | **5.80** | **5.80** | 100.0 | PBDR | P | Patterson C, 2012 | [3] |
| **Thailand** | |  |  |  |  |  |  |  |  |
|  | Northeastern | 1996_2005 | **0.58** | **0.58** | NA | MBR | H | Panamonta O, 2011 | [54] |
| **Tunisia** | |  |  |  |  |  |  |  |  |
|  | Beja, Monastir, Gafsa | 1990_1994 | **7.40** | **6.69** | 96.0 | PBDR | P | Ben Khalifa F, 1997 | [55] |
|  | Kairoan | 1991_1993 | **7.40** | **7.60** | NA | PBDR | P or H | Diamond, 2006 | [1] |
|  | Beja | 1990_1999 | **7.40** | **7.70** | NA | PBDR | P or H | Diamond, 2006 | [1] |
|  | Gafsa | 1990_1999 | **7.40** | **8.50** | NA | PBDR | P or H | Diamond, 2006 | [1] |
|  | Monastir | 1990_1999 | **7.40** | **5.80** | NA | PBDR | P or H | Diamond, 2006 | [1] |
| **Ukraine** | |  |  |  |  |  |  |  |  |
|  | NW | 1985_1992 | **8.10** | **8.10** | NA | OPD | NA | Timchenko O I, 1996 | [56] |
| **United Kingdom** | |  |  |  |  |  |  |  |  |
|  | NW | 1991_2008 | **19.32** | **19.32** | NA | PBDR | P | Imkampe AK, 2011 | [57] |
| **United Republic of Tanzania** | | |  |  |  |  |  |  |  |
|  | Dar es Salaam | 1982_1991 | **0.92** | **0.92** | NA | MBR | P | Swai A B, 1993 | [58] |
| **United States of America** | |  |  |  |  |  |  |  |  |
|  | Seven areas (Population: African American Young) | 2002_2005 | **14.01** | **17.53** | NA | PBDR | P | Bell R, 2009 | [59] |
|  | Seven areas (Population: Asian-Pacific Islander Young) | 2002_2005 | **14.01** | **7.30** | NA | PBDR | P | Bell R, 2009 | [59] |
|  | Seven areas (Population: Hispanic Young) | 2002_2005 | **14.01** | **15.60** | NA | PBDR | P | Bell R, 2009 | [59] |
|  | Seven areas (Population: Navajo Young) | 2002_2005 | **14.01** | **2.13** | NA | PBDR | P | Bell R, 2009 | [59] |
|  | Seven areas (Population: Non-Hispanic White Young) | 2002_2005 | **14.01** | **27.47** | NA | PBDR | P | Bell R, 2009 | [59] |
| **Uruguay** | |  |  |  |  |  |  |  |  |
|  | Montevideo | 1992_1992 | **8.30** | **8.30** | 97.0 | PBDR | P or H | Diamond, 2006 | [1] |
| **Uzbekistan** | |  |  |  |  |  |  |  |  |
|  | NW | 2000_2000 | **1.20** | **1.20** | NA | OPD | NA | Rakhimova G G N, 2002 | [60] |
| **Venezuela (Bolivarian Republic of)** | | |  |  |  |  |  |  |  |
|  | Caracas | 1990_1994 | **0.10** | **0.10** | NA | PBDR | P or H | Diamond, 2006 | [1] |

**References**

1. The DIAMOND Project Group (2006) Incidence and trends of childhood Type 1 diabetes worldwide 1990-1999. The DIAMOND project Group. Diabet Med 23: 857-866.

2. Catanzariti L, Faulks K, Moon L, Waters AM, Flack J, et al. (2009) Australia's national trends in the incidence of Type 1 diabetes in 0-14-year-olds, 2000-2006. Diabet Med 26: 596-601.

3. Patterson CC, Gyurus E, Rosenbauer J, Cinek O, Neu A, et al. (2012) Trends in childhood type 1 diabetes incidence in Europe during 1989-2008: evidence of non-uniformity over time in rates of increase. Diabetologia 55: 2142-2147.

4. Peter SA, Johnson R, Taylor C, Hanna A, Roberts P, et al. (2005) The incidence and prevalence of type-1 diabetes mellitus. J Natl Med Assoc 97: 250-252.

5. Zalutskaya A, Bornstein SR, Mokhort T, Garmaev D (2004) Did the Chernobyl incident cause an increase in Type 1 diabetes mellitus incidence in children and adolescents? Diabetologia 47: 147-148.

6. Tahirovic H, Toromanovic A (2007) Incidence of type 1 diabetes mellitus in children in Tuzla Canton between 1995 and 2004. Eur J Pediatr 166: 491-492.

7. Radosevic B, Bukara-Radujkovic G, Miljkovic V, Pejicic S, Bratina N, et al. (2013) The incidence of type 1 diabetes in Republic of Srpska (Bosnia and Herzegovina) and Slovenia in the period 1998-2010. Pediatr Diabetes 14: 273-279.

8. Negrato CA, Dias JP, Teixeira MF, Dias A, Salgado MH, et al. (2010) Temporal trends in incidence of Type 1 diabetes between 1986 and 2006 in Brazil. J Endocrinol Invest 33: 373-377.

9. EURODIAB ACE Study Group (2000) Variation and trends in incidence of childhood diabetes in Europe. Lancet 355: 873-876.

10. Ehrlich RM, Walsh LJ, Falk JA, Middleton PJ, Simpson NE (1982) The incidence of type 1 (insulin-dependent) diabetes in Toronto. Diabetologia 22: 289-291.

11. Blanchard JF, Dean H, Anderson K, Wajda A, Ludwig S, et al. (1997) Incidence and prevalence of diabetes in children aged 0-14 years in Manitoba, Canada, 1985-1993. Diabetes Care 20: 512-515.

12. Legault L, Polychronakos C (2006) Annual incidence of type 1 diabetes in Quebec between 1989-2000 in children. Clin Invest Med 29: 10-13.

13. Newhook LA, Penney S, Fiander J, Dowden J (2012) Recent incidence of type 1 diabetes mellitus in children 0-14 years in Newfoundland and Labrador, Canada climbs to over 45/100,000: a retrospective time trend study. BMC Res Notes 5: 628.

14. Larenas G, Montecinos A, Manosalva M, Barthou M, Vidal T (1996) Incidence of insulin-dependent diabetes mellitus in the IX region of Chile: ethnic differences. Diabetes Res Clin Pract 34 Suppl: S147-151.

15. Torres-Aviles F, Carrasco E, Icaza G, Perez-Bravo F (2010) Clustering of cases of type 1 diabetes in high socioeconomic communes in Santiago de Chile: spatio-temporal and geographical analysis. Acta Diabetol 47: 251-257.

16. Yang Z, Long X, Shen J, Liu D, Dorman JS, et al. (2005) Epidemics of type 1 diabetes in China. Pediatr Diabetes 6: 122-128.

17. Stipancic G, La Grasta Sabolic L, Pozgaj Sepec M, Radica A, Skrabic V, et al. (2012) Regional differences in incidence and clinical presentation of type 1 diabetes in children aged under 15 years in Croatia. Croat Med J 53: 141-148.

18. Skordis N, Efstathiou E, Kyriakides TC, Savvidou A, Savva SC, et al. (2012) Epidemiology of type 1 diabetes mellitus in Cyprus: rising incidence at the dawn of the 21st century. Hormones (Athens) 11: 86-93.

19. Arab M (1992) Diabetes mellitus in Egypt. World Health Stat Q 45: 334-337.

20. Teeaar T, Liivak N, Heilman K, Kool P, Sor R, et al. (2010) Increasing incidence of childhood-onset type 1 diabetes mellitus among Estonian children in 1999-2006. Time trend analysis 1983-2006. Pediatr Diabetes 11: 107-110.

21. Alemu S, Dessie A, Seid E, Bard E, Lee PT, et al. (2009) Insulin-requiring diabetes in rural Ethiopia: should we reopen the case for malnutrition-related diabetes? Diabetologia 52: 1842-1845.

22. Harjutsalo V, Sund R, Knip M, Groop PH (2013) Incidence of type 1 diabetes in Finland. JAMA 310: 427-428.

23. Mauny F, Grandmottet M, Lestradet C, Guitard J, Crenn D, et al. (2005) Increasing trend of childhood type 1 diabetes in Franche-Comte (France): analysis of age and period effects from 1980 to 1998. Eur J Epidemiol 20: 325-329.

24. Barat P, Valade A, Brosselin P, Alberti C, Maurice-Tison S, et al. (2008) The growing incidence of type 1 diabetes in children: the 17-year French experience in Aquitaine. Diabetes Metab 34: 601-605.

25. Amirkhanashvili K, Bikashvili N, Lapanashvili T, Metrevele D, Koplatadze K, et al. (2000) Epidemiology of the diabetes type 1 in Georgian children population, 1990-1999 study. Diabetologia 43 (suppl 1): A93 (365). Ref Type: Abstract. [Cited in the International Diabetes Federation, 2009].

26. Patterson CC, Dahlquist GG, Gyurus E, Green A, Soltesz G (2009) Incidence trends for childhood type 1 diabetes in Europe during 1989-2003 and predicted new cases 2005-20: a multicentre prospective registration study. Lancet 373: 2027-2033.

27. Dacou-Voutetakis C, Karavanaki K, Tsoka-Gennatas H (1995) National data on the epidemiology of IDDM in Greece. Cases diagnosed in 1992. Hellenic Epidemiology Study Group. Diabetes Care 18: 552-554.

28. Ramachandran A, Snehalatha C, Krishnaswamy CV (1996) Incidence of IDDM in children in urban population in southern India. Madras IDDM Registry Group Madras, South India. Diabetes Res Clin Pract 34: 79-82.

29. Pishdad GR (2005) Low incidence of type 1 diabetes in Iran. Diabetes Care 28: 927-928.

30. Roche EF, Menon A, Gill D, Hoey HM (2002) Incidence of type 1 diabetes mellitis in children aged under 15 years in the Republic of Ireland. J Pediatr Endocrinol Metab 15: 1191-1194.

31. Koton S (2007) Incidence of type 1 diabetes mellitus in the 0- to 17-yr-old Israel population, 1997-2003. Pediatr Diabetes 8: 60-66.

32. Bruno G, Maule M, Merletti F, Novelli G, Falorni A, et al. (2010) Age-period-cohort analysis of 1990-2003 incidence time trends of childhood diabetes in Italy: the RIDI study. Diabetes 59: 2281-2287.

33. Kawasaki E, Matsuura N, Eguchi K (2006) Type 1 diabetes in Japan. Diabetologia 49: 828-836.

34. Ajlouni K, Qusous Y, Khawaldeh AK, Jaddou H, Batiehah A, et al. (1999) Incidence of insulin-dependent diabetes mellitus in Jordanian children aged 0-14 y during 1992-1996. Acta Paediatr Suppl 88: 11-13.

35. Formosa N, Calleja N, Torpiano J (2012) Incidence and modes of presentation of childhood type 1 diabetes mellitus in Malta between 2006 and 2010. Pediatr Diabetes 13: 484-488.

36. Gomez-Diaz RA, Perez-Perez G, Hernandez-Cuesta IT, Rodriguez-Garcia Jdel C, Guerrero-Lopez R, et al. (2012) Incidence of type 1 diabetes in Mexico: data from an institutional register 2000-2010. Diabetes Care 35: e77.

37. Van Wouwe JP, Verkerk PH, Mattiazzo GF, El Mokadem N, HiraSing RA (2002) Variation by ethnicity in incidence of diabetes type 1 and clinical condition at onset in the Netherlands. Eur J Pediatr 161: 559-560.

38. Campbell-Stokes PL, Taylor BJ (2005) Prospective incidence study of diabetes mellitus in New Zealand children aged 0 to 14 years. Diabetologia 48: 643-648.

39. Soliman AT, al-Salmi IS, Asfour MG (1996) Epidemiology of childhood insulin-dependent diabetes mellitus in the Sultanate of Oman. Diabet Med 13: 582-586.

40. Ogle GD, Lesley J, Sine P, McMaster P (2001) Type 1 diabetes mellitus in children in Papua New Guinea. P N G Med J 44: 96-100.

41. Jarosz-Chobot P, Polanska J, Szadkowska A, Kretowski A, Bandurska-Stankiewicz E, et al. (2011) Rapid increase in the incidence of type 1 diabetes in Polish children from 1989 to 2004, and predictions for 2010 to 2025. Diabetologia 54: 508-515.

42. Al-Zyoud M, Al Ali M, Rahim A, M. I (1997) Insulin dependant diabetes mellitus (IDDM) in children below 13 years of age in Qatar. Diabetes Insights: 4-10. [Cited in the International Diabetes Federation, 2009].

43. Serban V, Timar R, Dabelea D, Green A, McKinney P, et al. (2001) The epidemiology of childhood-onset type 1 diabetes mellitus in Romania. ONROCAD Study Group. National Romanian Organisation for the Care of Diabetic Children and Adolescents. J Pediatr Endocrinol Metab 14: 535-541.

44. Pronina EA, Petraikina EE, Antsiferov MB, Duchareva OV, Petrone A, et al. (2008) A 10-year (1996-2005) prospective study of the incidence of Type 1 diabetes in Moscow in the age group 0-14 years. Diabet Med 25: 956-959.

45. Kulaylat NA, Narchi H (2000) A twelve year study of the incidence of childhood type 1 diabetes mellitus in the Eastern Province of Saudi Arabia. J Pediatr Endocrinol Metab 13: 135-140.

46. Habeb AM, Al-Magamsi MS, Halabi S, Eid IM, Shalaby S, et al. (2011) High incidence of childhood type 1 diabetes in Al-Madinah, North West Saudi Arabia (2004-2009). Pediatr Diabetes 12: 676-681.

47. Vlajinac HD, Bojovic BM, Sipetic SB, Adanja BJ, Jarebinski MS, et al. (1995) Insulin dependent diabetes mellitus: incidence in childhood in Belgrade 1982-92. J Epidemiol Community Health 49: 107-108.

48. Lee WW, Ooi BC, Thai AC, Loke KY, Tan YT, et al. (1998) The incidence of IDDM in Singapore children. Singapore Med J 39: 359-362.

49. Serrano Rios M, Moy CS, Martin Serrano R, Minuesa Asensio A, de Tomas Labat ME, et al. (1990) Incidence of type 1 (insulin-dependent) diabetes mellitus in subjects 0-14 years of age in the Comunidad of Madrid, Spain. Diabetologia 33: 422-424.

50. Lora-Gomez RE, Morales-Perez FM, Arroyo-Diez FJ, Barquero-Romero J (2005) Incidence of Type 1 diabetes in children in Caceres, Spain, during 1988-1999. Diabetes Res Clin Pract 69: 169-174.

51. Morales-Perez FM, Barquero-Romero J, Perez-Miranda M (2000) Incidence of type I diabetes among children and young adults (0-29 years) in the province of Badajoz, Spain during 1992 to 1996. Acta Paediatr 89: 101-104.

52. Elamin A, Ghalib M, Eltayeb B, Tuvemo T (1997) High incidence of type 1 diabetes mellitus in Sudanese children, 1991-1995. Ann Saudi Med 17: 478-480.

53. Dahlquist GG, Nystrom L, Patterson CC (2011) Incidence of Type 1 Diabetes in Sweden Among Individuals Aged 0-34 Years, 1983-2007: An analysis of time trends. Diabetes Care 34: 1754-1759.

54. Panamonta O, Thamjaroen J, Panamonta M, Panamonta N, Suesirisawat C (2011) The rising incidence of type 1 diabetes in the northeastern part of Thailand. J Med Assoc Thai 94: 1447-1450.

55. Ben Khalifa F, Mekaouar A, Taktak S, Hamhoum M, Jebara H, et al. (1997) A five-year study of the incidence of insulin-dependent diabetes mellitus in young Tunisians (preliminary results). Diabetes Metab 23: 395-401.

56. Timchenko OI, Kozachok GS, Turos EI, Omel'chenko EM (1996) [The prevalence of diabetes mellitus in children of different regions of Ukraine]. Tsitol Genet 30: 70-73 [Cited in the International Diabetes Federation, 2009].

57. Imkampe AK, Gulliford MC (2011) Trends in Type 1 diabetes incidence in the UK in 0- to 14-year-olds and in 15- to 34-year-olds, 1991-2008. Diabet Med 28: 811-814.

58. Swai AB, Lutale JL, McLarty DG (1993) Prospective study of incidence of juvenile diabetes mellitus over 10 years in Dar es Salaam, Tanzania. BMJ 306: 1570-1572.

59. Bell RA, Mayer-Davis EJ, Beyer JW, D'Agostino RB, Jr., Lawrence JM, et al. (2009) Diabetes in non-Hispanic white youth: prevalence, incidence, and clinical characteristics: the SEARCH for Diabetes in Youth Study. Diabetes Care 32 Suppl 2: S102-111.

60. Rakhimova G G N, Ismailov S (2002) Prevalence of Type 1 diabetes mellitus and its vascular complications in childhood population in the Republic of Uzbekistan according to a national register. Diabetologia 45 A107. Ref Type: Abstract. [Cited in the International Diabetes Federation, 2009].
