# Supplementary material for: Covariation of the Incidence of Type 1 Diabetes with Country Characteristics Available in Public Databases
Source: PLoS One. 2015 Feb 23;10(2):e0118298. doi: 10.1371/journal.pone.0118298 (PMC4338253; doi:10.1371/journal.pone.0118298)
Supplement: S3 Table — CI, Confidence Intervals. (a) Age-standardized estimate; (b) per 100,000 individuals; (c) Coverage among 1-year-olds (%). (DOCX) [file pone.0118298.s006.docx]

| **Model** | **Code** | **Coefficients** | **Estimate** | **CI 2.5%** | **CI 97.5%** | **Std. Error** | **t-value** | ***p*-value** |
| --- | --- | --- | --- | --- | --- | --- | --- | --- |
| ***Climate and environment*** |  |  |  |  |  |  |  |  |
| Res. standard error: 7.834 on 73 DF; **Adjusted R2: 0.3519**; F-statistic: 14.76 on 3 and 73 DF, *p*-value: 1.324e-07 |  | (Intercept) | 18.113 | 13.071 | 23.155 | 2.530 | 7.160 | 0.000 |
|  | ***CE_10*** | CO2 emissions (metric tons per capita) | 0.462 | 0.189 | 0.735 | 0.137 | 3.371 | 0.001 |
|  | ***CE_16*** | Outdoor air pollution (Annual PM10 [µg/m3]) | -0.008 | -0.060 | 0.045 | 0.026 | -0.288 | 0.774 |
|  | ***CE_18*** | UV radiation (J/mt2, 2004) | -0.003 | -0.005 | -0.002 | 0.001 | -4.427 | 0.000 |
| ***Demography*** |  |  |  |  |  |  |  |  |
| Res. standard error: 7.948 on 75 DF; **Adjusted R2: 0.3274**; F-statistic: 10.61 on 4 and 75 DF, *p*-value: 7.2e-07; AIC=336.5 |  | (Intercept) | -12.909 | -79.608 | 11.928 | 6.690 | -1.930 | 0.057 |
|  | ***DD_19*** | Adolescent fertility rate (births per 1,000 women ages 15-19) | -0.121 | -0.199 | -0.019 | 0.043 | -2.776 | 0.007 |
|  | ***DD_21*** | Fertility rate, total (births per woman) | 4.071 | 0.956 | 8.011 | 1.716 | 2.373 | 0.020 |
|  | ***DD_27*** | Population ages 65 and above (% of total) | 0.744 | 0.181 | 1.125 | 0.217 | 3.430 | 0.001 |
|  | ***DD_33*** | Urban population (% of total population) | 0.147 | -0.032 | 0.237 | 0.049 | 2.986 | 0.004 |
| ***Economy factors*** |  |  |  |  |  |  |  |  |
| Res. standard error: 7.362 on 66 DF; **Adjusted R2: 0.4499**; F-statistic: 10.81 on 6 and 66 DF, *p*-value: 2.397e-08 |  | (Intercept) | -1.488 | -11.044 | 8.068 | 4.786 | -0.311 | 0.757 |
|  | ***ED_39*** | Energy use (kg of oil equivalent per capita) | 0.001 | 0.000 | 0.001 | 0.000 | 1.761 | 0.083 |
|  | ***ED_41*** | Mobile cellular subscriptions (per 100 people) | 0.105 | 0.039 | 0.172 | 0.033 | 3.151 | 0.002 |
|  | ***ED_36*** | Adjusted savings: education expenditure (% of GNI) | 1.131 | 0.052 | 2.209 | 0.540 | 2.092 | 0.040 |
|  | ***ED_37*** | Improved sanitation facilities (% of population with access) | -0.097 | -0.225 | 0.031 | 0.064 | -1.506 | 0.137 |
|  | ***EH_45*** | Health expenditure per capita, (constant 2005 international $) | 0.002 | 0.001 | 0.003 | 0.001 | 2.999 | 0.004 |
|  | ***EC_33*** | GDP growth (annual %) | -0.473 | -1.028 | 0.083 | 0.278 | -1.700 | 0.094 |
| ***Health conditions*** |  |  |  |  |  |  |  |  |
| Res. standard error: 7.23 on 65 DF; **Adjusted R2: 0.4604**; F-statistic: 9.776 on 7 and 65 DF, *p*-value: 2.949e-08 |  | (Intercept) | -105.151 | -163.500 | -46.802 | 29.216 | -3.599 | 0.001 |
|  | ***HD_71*** | Chronic respiratory diseases, deaths. (a)(b) Male | 0.074 | -0.018 | 0.166 | 0.046 | 1.615 | 0.111 |
|  | ***HI_59*** | Hepatitis B (HepB3) immunization (c) | -0.118 | -0.174 | -0.062 | 0.028 | -4.208 | 0.000 |
|  | ***HR_56*** | Total alcohol in liters of pure alcohol >15yr. | -0.194 | -0.640 | 0.251 | 0.223 | -0.872 | 0.387 |
|  | ***HR_47*** | Mean BMI (kg/m2) (a) Male >20 yr. | 3.319 | 1.411 | 5.228 | 0.956 | 3.474 | 0.001 |
|  | ***HR_51*** | Mean Total Cholesterol in mmol/l (a) Male >25yr. | 8.205 | -0.026 | 16.437 | 4.122 | 1.991 | 0.051 |
|  | ***HR_54*** | Preterm birth rate (per 100 live births) | -0.604 | -1.489 | 0.281 | 0.443 | -1.363 | 0.178 |
|  | ***HR_55*** | Prevalence of undernourishment (% of population) | 0.189 | -0.168 | 0.545 | 0.178 | 1.058 | 0.294 |
| ***Final Summary Model**** |  |  |  |  |  |  |  |  |
| Res. standard error: 6.84 on 70 DF; **Adjusted R2: 0.5114**; F-statistic: 14.26 on 6 and 70 DF, *p*-value: 1.539e-10; AIC=302.76 |  | (Intercept) | -25.240 | -55.934 | 5.460 | 15.390 | -1.640 | 0.106 |
|  | ***CE_18*** | UV radiation (J/mt2, 2004) | -0.002 | -0.003 | 0.000 | 0.001 | -1.952 | 0.055 |
|  | ***DD_21*** | Fertility rate, total (births per woman) | 2.181 | -0.430 | 4.793 | 1.309 | 1.666 | 0.100 |
|  | ***ED_41*** | Mobile cellular subscriptions (per 100 people) | 0.066 | 0.012 | 0.121 | 0.027 | 2.428 | 0.018 |
|  | ***EH_45*** | Health expenditure per capita (constant 2005 international $) | 0.001 | 0.000 | 0.003 | 0.001 | 2.401 | 0.019 |
|  | ***HI_59*** | Hepatitis B (HepB3) immunization (c) | -0.085 | -0.139 | -0.032 | 0.027 | -3.187 | 0.002 |
|  | ***HR_47*** | Mean BMI (kg/m2) (a) Male >20 yr. | 1.263 | 0.045 | 2.480 | 0.611 | 2.068 | 0.042 |
