# Supplementary material for: Covariation of the Incidence of Type 1 Diabetes with Country Characteristics Available in Public Databases
Source: PLoS One. 2015 Feb 23;10(2):e0118298. doi: 10.1371/journal.pone.0118298 (PMC4338253; doi:10.1371/journal.pone.0118298)
Supplement: S1 Protocol — International Prospective Register of Systematic Reviews. (PDF) [file pone.0118298.s007.pdf]

PROSPERO International prospective register of systematic reviews

Systematic review of the variation in incidence of Diabetes Type 1 by age and period worldwide (1975-2014)

Paula A Diaz, Alain Jacques Valleron

Citation

Paula A Diaz, Alain Jacques Valleron. Systematic review of the variation in incidence of Diabetes Type 1 by age and period worldwide (1975-2014). PROSPERO 2012:CRD42012002369 Available from http://www.crd.york.ac.uk/PROSPERO_REBRANDING/display_record.asp?ID=CRD42012002369

Review question(s)

Is early age at onset of type 1 diabetes responsible for the increase in incidence trends worldwide?

Searches

Searching MEDLINE, the National Library of Medicine's database through PubMed, Web of Knowledge and Google Scholar we intend to identify the journal articles published in English, French, Spanish, to include in this review.

Types of study to be included

We will include articles whose main subjects are epidemiological studies with reports of incidence of T1D from population-based data registers by year and age at diagnosis with out any restriction about region worldwide or publication date. The studies must follow the World Health Organization (1985 or 1999) or the American Diabetes Association (1997 or 2011) as criteria of diagnosis of T1D.

Condition or domain being studied

Type 1 diabetes. Epidemiology. Incidence. Age factors. Age groups. Age distribution.

Participants/ population

Inclusion criteria for incidence analysis:

1) The selection will include articles whose main subjects are epidemiological studies with reports of incidence of T1D from population-based data registers by year and age at diagnosis with out any restriction about or region worldwide or publication date.

2) Reported data are on children as well as adults.

Exclusion criteria for incidence analysis:

The literature search will finish removing all articles with at least one of the following six exclusion criteria:

1) if the main objective is not the study of incidence of T1D (e.g., genetic factors, complications, treatments).

2) if the study does not follow the World Health Organization (1985 or 1999) or the American Diabetes Association (1997 or 2011) as criteria of diagnosis of T1D.

3) if the study does not have any new data that had previously been reported in another article in a specific place and time.

4) if the study is not performed in general population.

5) if the study include data that are not from original studies.

6) if the study is a review or a general topic about T1D.

Intervention(s), exposure(s)

Incidence data of type 1 diabetes and age at onset by global region and country will be taken from the individual studies as are reported in the publications in text, tables or graphs, as well as year of publication, length of study, number of cases and population at risk.

Comparator(s)/ control

We will compare the incidence data of type 1 diabetes and age at onset, by global region and country according to incidence levels.

Outcome(s)

Primary outcomes

Incidence rates of type 1 diabetes in males, females, and both males and females. Note: Incidence rate is defined as the number of newly diagnosed cases per calendar year and 100,000 population at risk.

Age at onset and year of diagnosis of type 1 diabetes.

Secondary outcomes

None

None

Data extraction, (selection and coding)

In order to obtain the most comprehensive selection of references and to prevent important references being missed, a systematic, exhaustive, and iterative selection process to identify references that potentially satisfying the inclusion criteria will be conducted. The selection process will be performed by one author (P.D.) who will read the title, followed by the abstract and if justified, will read the full article. In addition, a concordance analyses between two review team members for references selection will be carried out.

Data are going to extracted into a standard data collection grid using Microsoft Excel version 8.0 software. Extracted data will include 3 categories as follows:

1) information about the study, namely: reference, first author, year of publication, world region, country, and city, period of study (start and end date in years), length of the study, entire age class of the patients, data source of the entire population, degree of ascertainment, data collection process (historic, prospective, both historic and prospective), and statistical analyses used in the study;

2) information about incidence, namely: incidence rates crude and standardized by global region, country, and city and by sexes, male, female and both sexes male and female, age at onset classes, percentage of annual increase in incidence with p value, and confidence intervals, number of incident cases, and population at risk; and

3) additional information, namely: remark notes about summary of the studies, standardization, source of extracted information (text, table or graphs).

Risk of bias (quality) assessment

The methodology for the selection process to identify references as well as extraction of data, and the analyses will be discussed with at least three team members of the Inserm Unit 707.

For each reference we will perform the assessment of the risk of bias by both study-level assessment and outcome-level assessment.

Any unusual patterns will be checked and discussed with others team members.

Strategy for data synthesis

We will provide a descriptive synthesis of the main results from the included references. Given the same outcome measure, we will pool the results in order to perform a meta-analyses and other analyses that will include statistical analyses such as simple linear regression to fit the temporal trend for each world region, a correlation coefficient to

evaluate associations between level of incidence and increase in incidence, and an estimating Age Period Cohort model to investigate trends in incidence of T1D worldwide and by regions.

Analysis of subgroups or subsets

Subgroup analyses will be done for countries according to the level of incidence defined by the World Health Organization in the Diamond Project*, such as very low, low, intermediate, high, and very high incidence countries. We also plan to do a subgroup analyses by age (0-4, 5-9, 10-14, 15-19, 20-24, 25-29, 30-34, 35-39, ... year of age) and by sexes, male, female and both male and female.

*Karvonen M, Viik-Kajander M, Moltchanova E, Libman I, LaPorte R, Tuomilehto J. Incidence of childhood type 1 diabetes worldwide. Diabetes Mondiale (DiaMond) Project Group. Diabetes Care. 2000;23(10):1516-26.

Dissemination plans

A paper will be submitted to a leading journal in this field. In addition, we plan to prepare and send one page summary report to lead researchers, clinicians, healthcare professionals, and organizations know more about tendency in T1D worldwide.

Contact details for further information

Paula A. Diaz

Unite de Santé Publique

INSERM Unit 707

Assistance Publique-Hôpitaux de Paris

Hôpital Saint-Antoine

184 rue du Faubourg Saint-Antoine

75012 Paris, France.

paulaandreadiaz@gmail.com, paula.diaz@u707.jussieu.fr

Organisational affiliation of the review

Unit of Public Health and INSERM Unit 707, Saint Antoine Hospital, 184 rue du Faubourg Saint-Antoine, 75012 Paris.

http://www.u707.jussieu.fr/www_u707/index_fr.php

Review team

Dr Paula A Diaz, MD Mg PhD Student At Paris VI Pierre et Marie Curie University, 75008 Paris and developing thesis at Unit of Public Health INSERM Unit 707, Saint Antoine Hospital, 184 rue du Faubourg Saint-Antoine, 75012 Paris, France.
Professor Alain Jacques Valleron, Professor and researcher at Unit of Public Health INSERM Unit 707, Saint Antoine Hospital, 184 rue du Faubourg Saint-Antoine, 75012 Paris, France and VI Pierre et Marie Curie University, 75008 Paris, France.

Collaborators

Professor Pierre-Yves Boëlle, Professor and researcher at Unit of Public Health INSERM Unit 707, Saint Antoine Hospital, 184 rue du Faubourg Saint-Antoine, 75012 Paris.

Details of any existing review of the same topic by the same authors

A previous study on this topic, the Onkamo's et al. literature review carried out in 1999 used published data from 37 studies in 27 countries. Onkamo P, Vaananen S, Karvonen M, Tuomilehto J. Worldwide increase in incidence of Type I diabetes - the analysis of the data on published incidence trends. Diabetologia. 1999; 42:1395-403.

Anticipated or actual start date
01 June 2011

Anticipated completion date
31 March 2014

Funding sources/sponsors
Department of science, technology and innovation - Colciencias, Republica de Colombia: Tution and academic fess for the admision to the Doctorate Program is covered by the award "Beca Francisco Jose de Caldas".

Université Pierre et Marie Curie and Inserm, France: host university and laboratory for the development of the academic program in Epidemiologie et Sciencies de l'Information Biomedicale.

Conflicts of interest
None known

Language
English

Country
France

Subject index terms status
Subject indexing assigned by CRD

Subject index terms
Diabetes Mellitus, Type 1; Humans

Stage of review
Ongoing

Date of registration in PROSPERO
23 May 2012

Date of publication of this revision
01 December 2014

Stage of review at time of this submission	Started	Completed
Preliminary searches	Yes	No
Piloting of the study selection process	Yes	No
Formal screening of search results against eligibility criteria	Yes	No
Data extraction	Yes	No
Risk of bias (quality) assessment	Yes	No
Data analysis	Yes	No

PROSPERO
International prospective register of systematic reviews
The information in this record has been provided by the named contact for this review. CRD has accepted this information in good faith and registered the review in PROSPERO. CRD bears no responsibility or liability for the content of this registration record, any associated files or external websites.
